# Psychosocial Impact of Dental Aesthetics Among Aboriginal and Torres Strait Islanders: Mixed‑Methods Study Using Psychosocial Impact of a Dental Aesthetics Questionnaire

**DOI:** 10.1016/j.identj.2025.109339

**Published:** 2025-12-15

**Authors:** Sonia Nath, Johanna Groundwater, Ria Aiyar, Joanne Hedges, Gina Guzzo, Kostas Kapellas, Alexander Pham, Lisa Jamieson

**Affiliations:** Indigenous Oral Health Unit, Australian Research Centre for Population Oral Health, Adelaide Dental School, The University of Adelaide, South Australia, Australia

**Keywords:** Aboriginal and Torres strait islander peoples, Aging, Dental aesthetics, Indigenous Australians, Oral health, Public health dentistry

## Abstract

**Objectives:**

Dental aesthetics play a vital role in social and emotional wellbeing (SEWB). This study examines the impact of dental aesthetics on SEWB among Aboriginal and Torres Strait Islander peoples.

**Methods:**

Participants completed a culturally adapted 8‑item Psychosocial Impact of Dental Aesthetics Questionnaire (PIDAQ) comprising four subscales: dental self‑confidence, social impact, psychological impact, and aesthetic concern. Sociodemographic characteristics, dental service use, and self‑rated health data were collected and used as covariates. Mean and standard deviation (SD) were calculated for the total PIDAQ and each subscale across covariate groups. Multivariable generalised linear models were fitted to estimate mean ratios (MRs) and 95% confidence intervals (CI) for PIDAQ outcomes. Semi-structured interviews (n = 136) explored how participants perceived the effects of dental aesthetics on self-esteem, social confidence, and SEWB, and were analysed using reflexive thematic analysis. The study was overseen by an Indigenous Oral Health Reference Group.

**Results:**

Among 187 participants, the mean total PIDAQ score was 23.93 (SD 7.93), indicating a moderate psychosocial impact of dental aesthetics. Problem‑driven dental attendance was consistently associated with greater burden across outcomes, including total PIDAQ (MR 1.19, 95% CI 1.06-1.34), dental self‑confidence (MR 1.20, 1.04-1.40), social impact (MR 1.21, 1.05-1.40), and aesthetic concern (MR 1.21, 1.08-1.33). Poor self-rated general health was linked to higher total scores (MR 1.12, 1.02-1.24) and greater aesthetic concern (MR 1.12, 1.00-1.28). Additionally, a last dental visit more than a year ago was also associated with higher aesthetic concern (MR 1.11, 1.00-1.23). Qualitative analyses showed how dental appearance influences confidence, social participation, intersects with racism/discrimination, and how aesthetic dental treatment can enhance SEWB.

**Conclusion:**

Dental aesthetics can significantly influence SEWB among Aboriginal and Torres Strait Islander people. Culturally safe, affordable dental care addressing clinical and psychosocial needs is essential.

## Introduction

Dental aesthetics, including the appearance, colour, alignment, and overall condition of teeth, play a critical role in shaping an individual’s social interactions, self-esteem, and emotional health. Across diverse contexts, dissatisfaction with dental appearance can significantly reduce quality of life, leading to social withdrawal and undermining confidence.^1^^,^^2^ In Australia, more than one-third (35.3%) of adults aged 17 years and older reported feeling uncomfortable with the appearance of their teeth and mouth.^3^^,^^4^ Poor dental aesthetics, such as missing, visibly carious, and maligned teeth, are not only personally distressing but also socially stigmatising.^5^^,^^6^ Their consequences are particularly evident in Aboriginal and Torres Strait Islander Communities, where oral aesthetics are intertwined with personal wellbeing, collective identity, and broader social and emotional wellbeing (SEWB).^7^ This can influence employment prospects, participation in social activities, and willingness to engage in community and cultural events.^8^^,^^9^ In this way, oral aesthetics can perpetuate cycles of exclusion and psychological distress, affecting not only individuals but also their families and communities, and undermining efforts to foster resilience and positive identity, all of which contribute to poorer oral health and SEWB among Aboriginal and Torres Strait Islander Australians.

Aboriginal and Torres Strait Islander Peoples’ views of SEWB include a broader concept of holistic health and life, highlighting culture, family, spirituality, land, and community.^10^ Colonisation and racism disrupt these connections, and the resulting losses of language, cultural practice, or place can be compounded by social signals such as dental appearance. It’s crucial to recognise that the ideals of dental aesthetics often stem from a Eurocentric perspective, which may not align with the dental characteristics and cultural values of Indigenous populations.^11^^,^^12^ The imposition of Western models of dental care, lack of cultural safety, and widespread socioeconomic disadvantage have all contributed to persistent disparities in both clinical oral health outcomes and subjective wellbeing.^7^^,^^13^, ^14^, ^15^ Furthermore, racism can contribute to psychological distress, eroding self-worth and community trust, and deepening barriers to seeking and receiving care. Structural racism manifests in reduced access to dental care, lower-quality treatment, and mistrust between Aboriginal patients and healthcare providers.^6^^,^^16^

Despite widely acknowledged links between dental aesthetics and psychosocial wellbeing, the specific impact of dental appearance on Aboriginal and Torres Strait Islander adults remains underexplored.^17^ While there is limited research available on how physical appearance affects SEWB, there are even fewer studies on associations with oral aesthetics.^1^^,^^2^^,^^18^ The Psychosocial Impact of Dental Aesthetics Questionnaire (PIDAQ) provides a method for quantifying these impacts, as it evaluates dental self-confidence, social impact, psychological impact, and aesthetic concerns. PIDAQ has been validated in multiple cultural and linguistic contexts, including European,^19^ Asian,^20^^,^^21^ and Australian^22^ populations. A critical gap persists as no studies to date have rigorously examined how dental aesthetics specifically influences SEWB among Aboriginal and Torres Strait Islander adults, nor have they evaluated the cultural appropriateness of PIDAQ in this population. As a result, it remains unclear whether existing measures meaningfully reflect Indigenous experiences of dental appearance and wellbeing.^17^ To address this gap, the aim of the study is to examine the psychological and social impact of dental aesthetics on Aboriginal and Torres Strait Islander adults by: (1) using a culturally adapted, short-form PIDAQ to quantify psychosocial effects, and (2) qualitatively exploring how perceptions of dental aesthetics influence SEWB and engagement in cultural and community life. We hypothesise that dental aesthetics will have a significant and multidimensional impact on SEWB. This study is significant because Aboriginal and Torres Strait Islander peoples experience distinct historical, social and cultural influences on health, which is shaped by colonisation, racism and resilience, that is not captured in mainstream research.

## Methods

The findings reported in this cross-sectional study are part of a broader mixed-methods study involving two phases.^13^ The first phase involved oral epidemiological examinations completed from July 2022 to December 2023, followed by a 12-month follow-up phase from March 2024 to March 2025. The study includes findings from the follow-up phase. Ethics approval for this study was granted by the Aboriginal Health Council of South Australia and the University of Adelaide Human Research Ethics Committee (#04-22-990). Informed consent was obtained from all study participants before their involvement in the research. This research was conducted in accordance with the CONSIDER statement^23^ for strengthening reporting of health research involving Aboriginal and Torres Strait Islander peoples (Supplemental 1).

### Positionality statement

The research prioritised Aboriginal and Torres Strait Islander leadership, governance structures, and active engagement in reflexive research practices.^24^ Study oversight was provided by an Indigenous Oral Health Unit Reference Group (IOHURG) and the Director of the Indigenous Oral Health Unit (JH), who offered guidance across all project aspects, including staff recruitment, participant engagement, data collection protocols, analytical procedures, and community feedback mechanisms. This research team also included seven non-Indigenous researchers, including the first author. As researchers, we acknowledge that our identities and backgrounds inevitably shape how we engage with and interpret research, particularly in work involving Aboriginal and Torres Strait Islander communities. We remain continually accountable to the communities whose lived realities and knowledges are at the heart of this research and commit to reflexivity throughout all stages of the research process.

### Study population and data collection

For participant selection, a convenience sample was used. Participants were recruited based on their accessibility and willingness to participate, initiated through two pathways. Firstly, through educational presentations to Aboriginal Community Controlled Health Organisations (ACCHO) staff about the project commencing. Staff referred eligible clients who were willing to participate in the program. Second, a snowball recruitment approach was employed, whereby enrolled participants were encouraged to invite family members and friends who met the study criteria to join the research. Inclusion criteria included adults aged 18 years or older who self-identified as Aboriginal and/or Torres Strait Islander and were willing to provide consent and participate at both baseline and 12-month follow-up. The exclusion criteria included individuals with cognitive and language barriers, as well as those not residing in South Australia during the study period. Randomisation was not possible as this was an observational study.

At baseline and follow-up, epidemiological examinations and a self-report questionnaire were completed in REDCap.^25^ Data collection and dental exams took place at participants’ homes, workplaces, community centres, or dental clinics, depending on what was most convenient and comfortable for each individual. For clinical/epidemiological procedures, dental examiners (SN, KK & AP) followed standard calibration protocols before commencing data collection, and periodic 6-monthly reliability checks were conducted throughout the study. Participants completed the questionnaire either independently or with assistance, as they preferred. Blinding was not employed due to the self-report nature of the psychosocial questionnaire. The key variables included age, gender, location, education level, household size, ownership of a healthcare card (HCC indicates social welfare income and eligibility for subsidised health services), and dental service utilisation. Dental access indicators included the time since the last dental visit (<1 year vs >1 year), avoidance of dental care due to cost (yes/no), and difficulty paying $100 for dental treatment (yes/no). Participants also provided information on self-rated general and oral health. Between baseline examinations and 12-month follow-up, participants were referred for dental treatment in private or public clinics, and the treatment costs were covered by the project. The research team booked appointments with dental clinics and organised transportation to and from clinics when requested by participants.

The psychosocial impact of dental aesthetics was assessed at follow-up using a culturally adapted version of the PIDAQ, which consisted of eight items across four domains: dental self-confidence, psychosocial impact, social impact, and aesthetic impact.^20^^,^^21^ Each item was rated on a 5-point Likert scale. To ensure cultural safety and acceptability, a few of the items were positively framed and reworded to strength-based statements (Supplemental 2). Reliability was assessed using Cronbach’s alpha, which confirmed strong internal consistency (α ≥ 0.90) across all domains (Supplemental 2). The total score was calculated by summing the responses from all eight items, resulting in a possible score range from 8 (no psychosocial impact) to 40 (maximum negative psychosocial impact of dental aesthetics). Higher scores indicate greater negative psychosocial impact related to dental appearance. The adapted questionnaire (Supplemental 3) underwent a review for face and content validity by IOHURG, followed by pilot testing with a small participant group (n = 10) to confirm clarity and cultural acceptability.

For the qualitative component, semi-structured interviews explored Aboriginal and Torres Strait Islander peoples’ experiences with dental care more broadly (eg, ‘can you tell us about your experiences with the dental care system?’); perceptions regarding their own oral health (eg, ‘how does the look of your teeth, gums and smile influence how you feel about yourself?’); and how their oral health connects with their overall SEWB (eg, ‘in what ways does your oral health impact your wellbeing?’). Interviews were completed in a location of participants’ choosing (eg, their home, a university research facility, a community centre, or via phone call), were audio-recorded, and transcribed.

### Data analysis

Descriptive statistics were used to summarise study participant characteristics using mean and standard deviation (SD) for continuous variables, and for categorical variables, frequencies with 95% confidence intervals (CI). For the PIDAQ total score and its four subscales (dental self-confidence, social impact, psychological impact, and aesthetic concern), bivariate analysis was conducted, with either a *t*-test applied for binary categories or one-way analysis of variance (ANOVA) used for multiple categories. Generalised linear models were used with a Gamma family and log link, and outcomes represented as mean ratios (MRs) and 95% CI for analysing the total PIDAQ score and four subscales. Covariates included age group, sex, residential location, education, household size, healthcare card ownership, time since last dental visit, reason for last dental visit, whether cost prevented care, difficulty in paying AUD$100 for dental treatment, and self‑rated general and oral health. Model adequacy was assessed using simple goodness‑of‑fit summaries (Pearson dispersion and deviance‑to‑degrees‑of‑freedom ratios) (Supplemental 4). The *P‑values* were adjusted using the Benjamini–Hochberg false discovery rate within each outcome. Missingness was profiled for all variables. Because the proportion of missing data was low, listwise deletion was applied for each model after complete-case selection of the relevant outcome and covariates. As a robustness check, a sensitivity analysis was conducted using ordinary least squares (OLS) for the total PIDAQ score, with HC3 robust standard errors, reporting adjusted coefficients along with 95% CIs. Statistical significance was set at *P* < .05. All analyses were conducted in R (version 4.2, 2024) using the *sandwich, lmtest,* and *broom* packages, and the codes are available at https://github.com/sonianath/PIDAQ.

Interview data were analysed using reflexive thematic analysis. NVivo (version 15) was used to support coding and theme generation across the six phases of analysis.^26^^,^^27^ The initial phase included data familiarisation, where the first authors repeatedly read transcripts alongside listening to the audio-recordings, taking initial notes. Codes were then generated at the semantic level from the transcripts, followed by compilation of codes for theme generation. Provisional themes were developed and reviewed by the research team. Final themes were subsequently reviewed, named, and defined, with extracts selected to develop the analytic narrative.

## Results

### Study population characteristics

There were 280 study participants, and 187 participants completed all the responses to the PIDAQ questionnaire and were included in the quantitative analysis (Table 1). Additionally, 136 participants consented to participate in qualitative interviews. Of 187 respondents, 17 did not answer one culturally sensitive item, ‘I don’t like my own teeth in photos’, which was judged missing‑not‑at‑random (MNAR). Primary scoring used subscale prorating when ≥75% of items were present. The mean age of participants was 45.78 years (SD = 15.5), and the majority were female (58.82%). Most participants resided in major cities (81.72%), while 13.27% lived in regional or remote areas. Nearly half (48.11%) had a tertiary qualification, and most lived in households of four people (72.38%). More than half had a HCC (54.01%), and dental service use was largely problem-driven, with 66.3% reporting their last dental visit was over 1 year ago, and 78.26% seeking care due to a problem rather than for a check-up, 63.24% avoiding visits due to expense, and 78.92% reporting difficulty paying $100 for treatment. Most participants rated their general health as good (65.40%), but 61.40% rated their oral health as poor.

**Table 1:** Characteristics of the study population (N = 187).

Variables	n	Percentage (95% CI)
Age category		
18-34 y	44	25.00 (19.1%, 32.0%)
35-54 y	80	44.02 (36.8%, 51.5%)
>55 y	60	30.98 (24.5%, 38.3%)
Missing	<5	NA
Age Mean (SD)	187	45.78 (15.50)*
Gender		
Female	110	58.82 (51.4%, 65.9%)
Male	77	41.18 (34.1%, 48.6%)
Indigenous identity		
Aboriginal	166	90.22 (84.8%, 93.9%)
Torres Strait Islander	1	0.54 (0.0%, 3.5%)
Both	6	3.26 (1.3%, 7.3%)
Other indigenous group	11	5.98 (3.2%, 10.7%)
Missing	<5	NA
Location		
Major cities	152	81.72 (75.2%, 86.8%)
Regional/Remote Australia	34	13.27 (13.2%, 24.8%)
Missing	<5	NA
Education		
High school or less	96	51.89 (44.5%, 59.2%)
Tertiary education	89	48.11 (40.8%, 55.5%)
Missing	<5	NA
Household size		
0-4	131	72.38 (65.2%, 78.6%)
>4	50	27.62 (21.4%, 34.8%)
Missing	6	NA
Healthcare card		
No	86	45.99 (38.7%, 53.4%)
Yes	101	54.01 (46.6%, 61.3%)
Missing	<5	NA
Last dental visit		
Less than 1 y ago	62	33.70 (27.0%, 41.1%)
More than 1 y ago	122	66.30 (58.9%, 73.0%)
Missing	<5	NA
Reason for last dental visit		
Check-up	40	21.74 (16.2%, 28.5%)
Problem	144	78.26 (71.5%, 83.8%)
Missing	<5	NA
Avoided dental visit due to cost		
No	68	36.76 (29.9%, 44.2%)
Yes	117	63.24 (55.8%, 70.1%)
Missing	<5	NA
Difficulty in paying $100 for dental treatment		
Hard	146	78.92 (72.2%, 84.4%)
Not hard	39	21.08 (15.6%, 27.8%)
Missing	2	NA
Self-rated general health		
Good	121	65.40 (58.0%, 72.1%
Poor	64	34.59 (27.9%, 42.0%)
Missing	<5	NA
Self-rated oral health		
Good	58	31.35 (24.9%, 38.6%)
Poor	127	61.4%, 75.1%)
Missing	<5	NA

⁎ The mean and standard deviation was calculated for age.

#### The psychosocial impact is higher among those with problem-driven dental visits, financial barriers, and poor self-rated oral health

Table 2 shows the mean PIDAQ scores for the four subscales across all the variables. The mean total PIDAQ score was 23.93 (SD 7.93), with subscales scored as: dental self-confidence 5.83 (SD 2.39), social impact 6.27 (SD 2.33), psychological impact 5.97 (SD 2.17), and aesthetic concern 5.85 (SD 2.01). Participants whose last dental visit was for a problem had significantly higher scores across all subscales (total PIDAQ: 24.99 vs 20.60 for check-up, *P* < .01). Those who avoided dental visits due to cost (24.82 vs 22.60, *P* = .08) or reported difficulty paying $100 for treatment (24.48 vs 22.12, *P* = .10) had higher mean scores. Poor self-rated general health (26.62 vs 22.56, *P* <.01) and poor self-rated oral health (25.33 vs 20.67, *P* <.01) were strongly associated with higher PIDAQ scores across all domains.

**Table 2:** Mean and standard deviation of PIDAQ scores across the four domain variables (N = 187).

Variable	Levels (n)	Total PIDAQ	Dental self confidence	Social impact	Psychological impact	Aesthetic concern
Total (n = 187)		23.93 (7.93)	5.83 (2.39)	6.27 (2.33)	5.97 (2.17)	5.85 (2.01)
Age category	18-34 y (44)	24.81 (8.42)	5.91 (2.37)	6.30 (2.31)	6.37 (2.35)	5.89 (2.12)
	35-54 y (80)	22.71 (8.01)	5.62 (2.46)	6.11 (2.39)	5.63 (2.14)	5.69 (2.11)
	>55 y (60)	24.85 (7.42)	6.07 (2.34)	6.46 (2.35)	6.09 (2.09)	6.20 (2.00)
	F value; *P-value*	1.60; .20	0.62; .53	0.36; .69	1.83; .16	0.84; .42
Gender	Female (110)	23.82 (8.03)	5.79 (2.38)	6.32 (2.35)	5.95 (2.22)	5.83 (2.05)
	Male (77)	24.01 (8.03)	5.90 (2.43)	6.21 (2.32)	6.01 (2.06)	6.00 (2.11)
	Estimate; *P-value*	0.32; .74	0.29; .76	−0.31; .75	0.21; .83	0.50; .61
Location	Major cities (152)	23.89 (7.91)	5.79 (3.37)	6.30 (2.31)	5.99 (2.15)	5.84 (2.06)
	Regional/Remote Australia (34)	24.44 (8.06)	6.09 (2.53)	6.26 (2.44)	6.00 (2.24)	6.16 (2.11)
	Estimate; *P-value*	−0.43; .66	−0.63; .53	0.06; .94	−0.01; .98	−0.77; .44
Education	High school or less (96)	23.89 (8.02)	5.90 (2.46)	6.23 (2.46)	5.86 (2.27)	5.96 (2.20)
	Tertiary education (89)	23.95 (7.76)	5.76 (2.35)	6.33 (2.22)	6.07 (2.09)	5.82 (1.95)
	Estimate; *P-value*	−0.05: .95	0.37; .71	−0.28; .77	−0.63; .52	0.43; .66
Household size	0-4 (131)	24.78 (7.95)	6.22 (2.40)	6.46 (2.38)	6.21 (2.21)	6.07 (2.12)
	>4 (50)	22.07 (7.41)	4.84 (2.06)	5.86 (2.13)	5.34 (2.08)	5.49 (1.97)
	Estimate; *P-value*	−1.99; .04*	−3.84; <.01*	−1.63; .10	−2.48; .01*	−1.61; .10
Healthcare card	No (86)	23.66 (7.47)	5.83 (2.19)	6.09 (2.20)	5.93 (2.01)	5.88 (2.05)
	Yes (101)	24.27 (8.12)	5.84 (2.56)	6.43 (2.44)	6.01 (2.32)	5.92 (2.09)
	Estimate; *P-value*	−0.49; .32	−0.04; .96	−0.97; .32	−0.25; .80	−0.12; .90
Last dental visit	Less than 1 y ago (62)	23.06 (7.59)	5.63 (2.46)	6.02 (2.11)	6.05 (2.12)	5.45 (1.89)
	More than 1 y ago (122)	24.39 (7.97)	5.97 (2.36)	6.44 (2.43)	5.92 (2.23)	6.10 (2.13)
	Estimate; *P-value*	−0.99; .31	−0.89; .37	−1.23; .22	0.38; .69	−1.92; .05*
Reason for last dental visit	Check-up (40)	20.6 (6.17)	4.70 (1.99)	5.30 (2.17)	5.15 (1.93)	4.94 (1.43)
	Problem (144)	24.99 (8.04)	6.16 (2.42)	6.56 (2.33)	6.19 (2.21)	6.19 (2.16)
	Estimate; *P-value*	3.44; <.01*	3.90; <.01*	3.18; <.01*	2.93; <.01*	4.00; <.01*
Avoided dental visit due to cost	No (117)	22.60 (7.86)	5.37 (2.28)	5.30 (2.17)	5.59 (2.21)	5.68 (2.16)
	Yes (68)	24.82 (7.77)	6.10 (2.44)	6.44 (2.34)	6.18 (2.15)	6.03 (2.03)
	Estimate; *P-value*	1.72; .08	2.05; .04*	1.22; .22	1.77; .07	1.00; .31
Difficulty in paying $100 for dental treatment	Hard (146)	24.48 (7.98)	5.94 (2.37)	6.38 (2.36)	6.09 (2.20)	6.02 (2.20)
	Not hard (39)	22.12 (7.17)	5.44 (2.43)	5.87 (2.27)	5.49 (2.09)	5.44 (1.50)
	Estimate; *P-value*	1.66; .10	1.11: .26	1.23; .22	1.58; .11	1.79; .07
Self-rated general health	Good (121)	22.56 (7.99)	5.42 (2.38)	5.88 (2.36)	5.54 (2.20)	5.46 (2.00)
	Poor (64)	26.62 (6.91)	6.61 (2.27)	7.03 (2.27)	6.77 (1.93)	6.73 (1.98)
	Estimate; *P-value*	−3.32; <.01*	−3.33; <.01*	−3.37; <.01*	−3.91; <.01*	−3.83; <.01*
Self-rated oral health	Good (58)	20.67 (7.50)	4.95 (2.39)	5.52 (2.24)	5.10 (2.19)	4.93 (1.76)
	Poor (127)	25.33 (7.62)	6.24 (2.31)	6.62 (2.32)	6.35 (2.08)	6.29 (2.08)
	Estimate; *P-value*	−3.52; <.01*	−3.43; <.01*	−3.07; <.01*	−3.66; <.01*	−4.18; <.01*

PIDAQ, the Psychosocial Impact of Dental Aesthetics Questionnaire.

PIDAQ consisted of 8 items. Each item is rated on a 5-point Likert scale: 1 (‘Strongly agree’) to 5 (‘Strongly disagree’). The total score was calculated by summing the responses from all 8 items.

For PIDAQ, the score ranges from 8 (no psychosocial impact) to 40 (maximum negative psychosocial impact of dental aesthetics), and for the subscales, it ranges from 2 (no impact) to 10 (maximum impact).

The scores reported here represent the mean and standard deviation.

⁎ *t*-test or ANOVA was calculated with *P* < .05 considered significant.

In multivariable generalised linear models (Table 3), participants who reported visiting the dentist because of a problem rather than a check-up had significantly higher scores (total PIDAQ: MR 1.19, 95% CI 1.06-1.34, *P* = .01; dental self-confidence: MR 1.20, 1.04-1.40, *P* = .03; social impact: MR 1.21, 1.05-1.40, *P* = .04; psychological impact: MR 1.15, 1.00-1.32, *P* = .11; aesthetic concern: MR 1.21, 1.08-1.33, *P* = .05). Poor self-rated general health was associated with higher total (MR 1.12, 1.02-1.24, *P* = .05) and aesthetic concern scores (MR 1.12, 1.00-1.28, *P* = .02), with suggestive increases in the social and psychological domains. A last dental visit more than 1 year ago was associated with higher aesthetic concerns (MR 1.11, 1.00-1.23, *P* = .05). Poor self-rated oral health was associated with higher total (MR 1.13, 1.00-1.27, *P* = .09), self-confidence (MR 1.15, 1.00-1.33, *P* = .09), psychological (MR 1.15, 1.00-1.32, *P* = .11), and aesthetic concern (MR 1.15, 1.01-1.31, *P* = .05) scores. Compared to those aged 18 to 34 years, individuals aged 35 to 54 years had a lower total impact (MR 0.86, 0.77-0.96, *P* = .05), lower psychological impact (MR 0.82, 0.72-0.93, *P* < .01), and lower aesthetic concern (MR 0.87, 0.78-0.97, *P* = .05). Larger households (>4) had lower dental self-confidence (MR 0.79, 0.69-0.90, *P* < .01). Gender, education, healthcare card status, and residential location were not significantly associated with PIDAQ outcomes (*P* > .05 for all). Additionally, sensitivity analysis using OLS for the total PIDAQ score showed similar results, supporting robustness of the main analysis (Supplemental 5). Overall, the results show that the higher psychosocial impact of dental aesthetics was significantly associated with financial barriers, problem-driven dental visits, and poor self-perceived health.

**Table 3:** Associations between sociodemographic and dental behaviour factors and PIDAQ total and subscale scores estimated using Generalised Linear Models with Gamma family and log link (N = 187).

Variable	Levels	Model 1 PIDAQ			Model 2 dental self confidence			Model 3 social impact			Model 4 psychological impact			Model 5 aesthetic concern		
		MR (LCI -UCI)	*P*-value	*P*-value adj	MR (LCI -UCI)	*P*-value	*P*-value adj	MR (LCI -UCI)	*P*-value	*P*-value adj	MR (LCI -UCI)	*P*-value	*P*-value adj	MR (LCI -UCI)	*P*-value	*P*-value adj
Age category (ref = 18-34 y)	35-54 y	0.86 (0.77-0.96)	.01*	.05*	0.86 (0.75- 0.99)	.04*	.09	0.92 (0.79-1.03)	.16	.38	0.82 (0.72-0.93)	<.01*	<.01*	0.87 (0.78-0.97)	.01*	.05*
	> 55 y	0.95 (0.84-1.06)	.39	.46	0.94 (0.82-1.09)	.46	.58	0.99 (0.86-1.14)	.99	.99	0.92 (0.81 -1.05)	.22	.35	0.93 (0.83-1.05)	.27	.47
Gender (ref = Male)	Female	0.99 (0.90-1.09)	.88	.88	1.02 (0.91-1.14)	.68	.68	1.02 (0.91 -1.14)	.72	.77	0.95 (0.85-1.06)	.37	.52	0.97 (0.88-1.08)	.63	.71
Location (ref = Metro)	Regional/Remote	1.08 (0.96-1.22)	.16	.27	1.14 (0.98-1.32)	.07	.15	1.07 (0.93 -1.23)	.32	.50	1.02 (0.88-1.18)	.76	.76	1.11 (0.97-1.27)	.10	.20
Education (ref = Tertiary education)	High school or less	0.97 (0.80-1.01)	.61	.66	1.02 (0.91-1.15)	.58	.68	0.95 (0.84 -1.05)	.35	.50	0.95 (0.85-1.05)	.41	.53	0.98 (0.88-1.08)	.62	.71
Household size (ref = 0-4)	>4	0.90 (0.82- 1.04)	.08	.18	0.79 (0.69-0.90)	<.0*1	<.01*	0.95 (0.83 –1.08)	.47	.60	0.91 (0.80-1.04)	.19	.34	0.94 (0.83-1.07)	.35	.55
Healthcare card (ref = No)	Yes	1.03 (0.95-1.13)	.40	.46	1.02 (0.91-1.14)	.64	.68	1.07 (0.96-1.19)	.16	.38	1.02 (0.92-1.13)	.62	.71	1.01 (0.91-1.12)	.70	.71
Last dental visit (ref = Less than 1 y ago)	More than 1 y ago	1.06 (0.97-1.16)	.17	.27	1.09 (0.97-1.22)	.11	.19	1.07 (0.96-1.19)	.16	.38	0.97 (0.86-1.08)	.66	.71	1.11 (1.00-1.23)	.01*	.05*
Reason for last dental visit (ref = Check-up)	Problem	1.19 (1.06 -1.34)	<.01*	.01*	1.20 (1.04-1.40)	<.01*	.03*	1.21 (1.05-1.40)	<.01*	.04*	1.15 (1.00-1.32 0	.03*	.11	1.21 (1.08 -1.33)	<.01*	.05*
Avoided dental visit due to cost (ref = No)	Yes	1.07 (0.97-1.18)	.13	.26	1.13 (1.01- 1.28)	.03*	.09	1.06 (0.94-1.20)	.26	.47	1.07 (0.96-1.20)	.17	.34	1.04 (0.93 -1.16)	.50	.71
Difficulty in paying $100 for dental treatment (ref = Not hard)	Hard	1.07 (0.97- 1.18)	.33	.46	1.06 (0.91-1.23)	.41	.58	1.04 (0.91 -1.20)	.52	.61	1.09 (0.96-1.25)	.16	.34	1.02 (0.92 -1.14)	.71	.71
Self-rated general health (ref = Good)	Poor	1.12 (1.02-1.24)	.01*	.05*	1.09 (0.97-1.23)	.13	.20	1.12 (1.00-1.26)	.04*	.22	1.15 (1.01-1.25)	.02*	.11	1.12 (1.00-1.28)	<.01*	.02*
Self-rated oral health (ref = Good)	Poor	1.13 (1.0-1.27)	.03*	.09	1.15 (1.00-1.33)	.04*	.09	1.08 (0.94 (1.25)	.22	.44	1.15 (1.00-1.32)	.03*	.11	1.15 (1.01-1.31)	.02*	.05*

LCI, lower confidence interval; MR, mean ratio; PIDAQ, the Psychosocial Impact of Dental Aesthetics Questionnaire; *P*-value adj, *P*-value adjusted; UCI, upper confidence interval.

*P*-values adjusted for multiple comparisons using Benjamini–Hochberg (within-outcome).

A higher MR (>1) indicates a higher mean score compared to the reference. Each multivariable model adjusted for age category, sex, location, education, household size, healthcare card, time since last dental visit, reason for last dental visit, whether cost prevented care, difficulty in paying AUD$100 for dental treatment, self-rated general health, and self-rated oral health.

Reference levels are specified in the brackets.

Missing observations were excluded via complete‑case analysis.

⁎ *P-value* < .05 was considered significant.

### Qualitative findings

Four themes were generated from the interview data: (1) Perceived effects of dental aesthetics on self-worth, confidence and SEWB; (2) The role of dental aesthetics in social participation and community belonging; (3) Dental aesthetics intersect with experiences of racism and discrimination; and (4) Impact of aesthetic dental treatment on wellbeing.

#### Theme 1: ‘Smile is the most important thing you wear’. Perceived significance of dental aesthetics on self-worth, confidence, and SEWB

This theme describes participants’ overall general perceptions of appearance in relation to teeth, and how SEWB may play a role. Firstly, dental aesthetics, more specifically a smile, were highlighted as playing a significant role in overall appearance. Some participants emphasised the importance of a smile in lifting mood, fostering relationships, improving self-identity, building self-esteem and confidence:

*I think having really good teeth certainly gives you good self-esteem and stuff like that...I think it’s really lovely to have nice teeth and smile around like, you own the world (P245)*

Among Aboriginal and Torres Strait Islander people living in regional or remote areas, one participant highlighted that these communities were underserviced in terms of dental care, and that dental aesthetics was a concern for many, impacting their confidence:

*We lived in [remote] communities in the Territory…dental was a big thing where they didn’t have access, you’d have to drive all the way into Katherine [regional town]…it affects people’s confidence and when they laugh or they cover their mouth a lot…and I say, ‘Don’t do that! You got a lovely smile’. Yeah it affects confidence. (P341)*

For many participants, teeth were seen as key to appearance and wellbeing, emphasising the importance of a smile in boosting confidence, self-esteem, and health broadly:

*Oh it affects [wellbeing] a lot actually because your overall appearance, your teeth are the main source what people look at...I reckon your overall health when you feel happy and smile, makes your health, look at the way you present yourself, health wise. (P242)*

The importance of oral aesthetics on SEWB are significant. Self-esteem, confidence and social interactions are all influenced by what participants consider to be an ‘aesthetically pleasing smile’ (ie, no teeth visibly missing, white, straight teeth).

#### Theme 2: ‘I don’t like the way I look’. The role of dental aesthetics in social participation and belonging

This theme highlights how participants perceived the connection between the appearance of their teeth and the judgment of others. Participants often linked poor dental appearance to strong feelings of discomfort, embarrassment, and self-consciousness, with some becoming emotional when discussing the impact:

*Oh it affects me a lot like, I get emotional talking about this but it just, I don’t like the way I look. I don’t like my teeth, I don’t like none of it, and I know that it affects me a whole lot. (P217)*

Several participants also mentioned unsolicited comments from others about their teeth, noting that these interactions increased feelings of shame and reluctance to smile or speak openly:

*I haven’t seen people for a long time and I meet up with them and I’m happy to see them and I forget my smile and they’re just kind of thinking “Well what’s happened here?” (P011)*

Life circumstances such as periods of depression, relationship breakdowns, economic pressures, or other hardships were described as contributing to neglect of oral hygiene over time or the result of being unable to seek appropriate oral care when required. In such cases, participants reflected with regret on how these experiences had lasting consequences on their SEWB:

*…going through, like depression and stuff like that and relationship break-ups and it led to me not brushing my teeth and letting them go, which I’m very depressed about because I had great teeth. (P17)*

These narratives show that the emotional impact of these visible differences, particularly when judged against dominant Western ideals of a ‘straight and white’ smile, can lead to changes in behaviour such as avoiding smiling, covering one’s mouth, or withdrawing socially. Overall, the appearance of discoloured, misaligned, or missing teeth can trigger shame, self-consciousness, and reduced social participation, ultimately influencing SEWB. The visibility of these differences means they are not only a personal health matter, but also a source of social judgment and, for some, are related to experiences of stigma or bullying.

#### Theme 3: ‘Totally discriminated in the healthcare system*.*’ Dental aesthetics intersect with experiences of racism and discrimination

Participants shared experiences of racism and discrimination that were worsened by ideas that people held around dental appearance (to have white, straight and clean teeth). Many participants identified discrimination stemming from stereotyping based on appearance bias. Some participants described being perceived as high consumers of alcohol, heavy cigarette smokers or illicit drug users:

*When you get discriminated against because you’re skinny, you got no teeth, you’re automatically labelled or they assume that you’re a bad person, you’re a junkie, you’re an alcoholic. (P409)*

Similarly, another participant highlighted how experiences of racism can be exacerbated based on preconceived notions that non-Indigenous people may have regarding their appearance.

*I’ve never smoked in my life, but I worry, okay maybe my teeth look a little discoloured and people think that, and then they’ll learn more about me, they’ll know I’m Aboriginal and go, “Oh that makes sense then”. So it’s a lot of that anxiety in community. (P349)*

This participant describes the implications dental aesthetics may have in relation to cultural background, highlighting the racism and discrimination that follows and leading to anxiety within the community. One participant described the undermining of their symptoms (unrelated to oral health) as a result of healthcare workers’ assumptions of their oral appearance:

*Totally discriminated in the healthcare system, majorly…They took me to the [Hospital] to do a CAT scan on my head…said, “It’s all in your head” and sent me home. I was crying that hard…it was terrible, I’ve never ever been treated like that in my life. And I believe it’s a lot to do with my teeth as well, people look at me and they make their own assumptions. (P17)*

Similarly, parents recounted discrimination when taking their children to the dentist. As this participant below describes, one practitioner assumed (from Aboriginal and Torres Strait Islander status) that concerns with their children’s teeth were only related to diet, rather than other multifactorial causes for dental decay:

*‘I think the kids have [experienced judgement]…they [dentists] just assumed ‘cause they didn’t know me, it’s ‘cause I’m feeding them sugar and lollies and things or soft drinks, and that was never, ever, ever the case… there certainly has been judgement from that respect for the kids… (P435)*

Stereotyping on the part of practitioners and the broader community based on dental aesthetics and knowledge of Aboriginal and Torres Strait Islander background was a key issue. This theme highlights the ways in which perceptions around optimal dental aesthetics intersect with racism and discrimination.

#### Theme 4: ‘I actually feel really good’. Impact of aesthetic dental treatment on wellbeing

When dental treatment improves dental aesthetics, both preventatively and restoratively, participants discussed that it improved their SEWB. Many participants identified feeling happier and having improved confidence. One participant suggested a professional cleaning and the addition of a tooth added to a denture was enough to strengthen their SEWB:

*So they [dentist] just cleaned a few bits of teeth and that up, and with that one tooth that they had to put on the denture part. I love it. I love it. Makes you feel more happy. (P259)*

Participants who underwent restorative aesthetic treatment were pleased by the transformation it had on their ability to be present within social situations without feeling self-conscious:

*Yes it [dental treatment] was like way more emotional than I thought it would be, like I actually shed a tear. Like it was just, yeah, really good to be able to smile and not be so self-conscious. (P223)*

The aesthetic improvement for many participants promoted confidence to speak more, smile more, and eat in public. One participant reflected on peers commenting on the improvement of his smile and overall persona post the dental treatment:

*Now I actually feel really good, now I’ve got a plate in place. Now I can actually eat proper, I can eat steak [laughs] before I couldn’t. So feel good with myself, a lot of people like, “Oh stop smiling at me, it’s too bright!” [laughs]. (P98)*

Access to dental treatment within this study also promoted and reinforced the benefits of oral hygiene practices at home to maintain healthy teeth:

*Before I wasn’t brushing as often as I, but now I feel like since going to the dentist, especially having the clean done, I, you know, I brush my teeth morning and night, and I floss. (P145)*

Dental treatment influenced SEWB by improving confidence, self-esteem, and social interaction. Furthermore, dental appointments are supported with oral health promotion to maintain aesthetically pleasing oral health.

## Discussion

This mixed-methods study was the first to apply a culturally adapted short-form PIDAQ, in combination with qualitative interviews, to capture the intersection of dental aesthetics and SEWB among Aboriginal and Torres Strait Islander adults. Unlike previous work, which has relied mainly on oral health quality-of-life instruments^17^ or studied non-Indigenous populations,^28^, ^29^, ^30^ this research uniquely quantifies psychosocial impacts using a tool reviewed for cultural safety. The quantitative results indicate that the psychosocial burden of poor dental aesthetics was most evident among participants with problem-driven dental visits, financial barriers to care, and poor self-rated oral and general health. The qualitative analysis supported these findings, showing that oral aesthetics impacted confidence, self-worth, and relationships.

### The role of dental aesthetics in SEWB

The PIDAQ analysis revealed that participants with poor self-rated oral health experienced significantly greater psychosocial impacts, and this is consistent with the findings from Wahab et al^31^ The impact of dental aesthetics on SEWB may be influenced by multiple interconnected pathways through behavioural, biological, and psychosocial factors. Stigma or embarrassment about poor dental appearance can cause people to avoid social, occupational, and cultural activities, leading to isolation from important networks and opportunities for cultural growth participation.^5^ Participants described smiling as a source of identity and approachability, with poor oral aesthetics limiting confidence, social participation, and engagement in cultural events. Previous research highlights that smiling has a positive influence on both psychosocial and physical health, enhancing stress recovery and social relationships.^11^^,^^22^^,^^32^^,^^33^ From a biological perspective, poor oral health, such as untreated caries or periodontal disease and may cause chronic pain, inflammation, and nutritional compromises, contributing to heightened stress and poorer mental health outcomes.^4^^,^^34^ Psychosocially, the internalisation of Western ideals of dental aesthetics, which are reinforced by repeated exposure to racism and exclusion, can erode self-esteem and deepen feelings of shame, particularly among young people who may experience bullying or negative social comparison.^5^^,^^6^^,^^35^ The dominant Western ideas of dental aesthetics, which often define the ideal smile as having straight, white, and evenly spaced teeth, are promoted through media, advertising, and mainstream dental practice.^36^ Recent work further highlights that shame and social withdrawal associated with dental aesthetics not only influence behavioural avoidance but also have a direct impact on oral hygiene practices and patterns of care-seeking, often perpetuating a cycle of poor oral and systemic health.

### Financial barriers, racism and dental aesthetics

Problem-driven dental visits were consistently associated with poorer psychosocial outcomes. These patterns reveal how financial constraints prevent timely preventive care, leading to deteriorating dental aesthetics that compound feelings of shame and social withdrawal. Participants described cycles in which poor oral appearance led to avoidance of social situations, which further isolated them from community support and cultural activities central to SEWB. This suggests that addressing dental aesthetics should not be viewed as cosmetic or secondary, but rather as a means of restoring dignity, confidence, and social participation. Data from the Australian survey^4^ consistently show that poorer oral health status and greater aesthetic dissatisfaction are strongly correlated with social disadvantage, infrequent dental attendance, lower income, and being Indigenous or living in regional/remote locations. Multivariable modelling shows that higher levels of psychosocial impact from dental aesthetics (measured by the PIDAQ) are almost twice as high among Australians with significant oral impacts on daily life compared to those without.^31^

Literature shows that Aboriginal and Torres Strait Islander people are more likely to have inadequate dentition, experience toothache, attend for dental problems rather than check-ups, and have higher rates of decayed, missing, or filled teeth compared to non-Indigenous peers.^14^^,^^15^ Participants from this study reported how missing or discoloured teeth reinforce feelings of shame, stigma, and social withdrawal, which were also linked to experiences of racism and discrimination. These experiences echo broader literature on structural racism in oral health care delivery, which highlights mistrust and unequal treatment as barriers to care.^6^^,^^14^^,^^37^ These findings indicate that dental aesthetics function is not just a personal health concern, but also a social signal intersecting with colonial histories, racism, and exclusion.^16^ Our study also reported that younger participants experienced greater psychological impacts, with accounts of bullying and social exclusion during formative years. These findings align with those of other researchers who have reported age differences in perception across different age groups, with younger adults being more critical.^11^^,^^38^

After receiving aesthetic dental treatment, the study participants reported an improvement in SEWB. Dental clinicians are encouraged to emphasise preventive care through regular check-ups, helping to address issues before they become more complex and negatively impact wellbeing.^39^ Addressing and discussing financial barriers transparently can help patients access appropriate care without stigma or delay. Patients can also be educated about the connection between SEWB and dental aesthetics to promote positive oral health behaviours.

### Strengths and limitations

A key strength of this study is its mixed-methods design, which combines quantitative analysis using the culturally adapted PIDAQ with large-scale qualitative interview data. However, limitations include: (1) cross-sectional design, which prevents causal inference between dental aesthetics and SEWB; (2) convenience sample from South Australia, which may limit generalisability and introduce potential sampling bias; (3) the self-reported data may lead to recall and reporting bias; (4) self-selection bias as those with strong views may have been overrepresented; (5) lack of longitudinal and behavioural data; (6) social desirability bias may have impacted the findings if participants provided socially acceptable answers. Future research should include intervention or longitudinal follow-up studies, using behavioural and biological markers to clarify pathways and outcomes.

### Clinical and policy implications

The study findings have significant implications for oral health policy, practice, and clinical implications. Providers should prioritise both functional and aesthetic outcomes, address financial and structural barriers, and create non-judgmental environments that validate Aboriginal perspectives on a healthy smile. Broadening the scope of public dental schemes to include coverage of evidence-based aesthetic treatments such as implants (especially for anterior tooth loss), orthodontics, and restorative procedures that impact SEWB. This may require increased funding to mainstream government and Aboriginal Community Controlled dental clinics, or responsive service planning that addresses these unmet needs. Aesthetic problems are a priority issue formally recognised by some government dental organisations that allow the bypassing of waitlists^40^; however, these can be adopted in other states and territories of Australia, as well as in Aboriginal and Torres Strait Islander dental health programs. The national and state oral health surveys should monitor access, demand, and outcomes for aesthetic dental treatments across population groups, enabling responsive, evidence-based policy adjustments.

Additionally, it is essential to address racism within healthcare settings and broader community contexts, as poor oral aesthetics can exacerbate experiences of discrimination. Engaging in strength-based conversations that acknowledge and respect each patient’s unique perceptions of a healthy and confident smile is crucial. Training the future oral health workforce on the provision of culturally safe dental care,^41^ the need for dental providers to broaden treatment outcomes to include aesthetics (from only functionality) to provide holistic, patient-centred care.

To support implementation, two practical actions are proposed: (1) establish immediate, culturally safe referral pathways through ACCHOs and local Indigenous oral health services; and (2) institute cost‑relief strategies (eg, fee waivers or vouchers prioritising high‑impact aesthetic needs such as anterior restorations).

## Conclusion

This mixed‑methods study demonstrates that dental aesthetics significantly influence the SEWB of Aboriginal and Torres Strait Islander Australian adults, particularly among those experiencing cost barriers, problem‑driven dental attendance, and poor self‑rated health. When interpreting these findings, it is essential to consider the study’s limitations. Clinically and from a public health perspective, dental aesthetics is not merely cosmetic; it is fundamental to restoring dignity, self‑determination, and social participation. Integrating aesthetic care into culturally safe, affordable, and accessible oral health services can tangibly improve SEWB, reduce social exclusion, and strengthen community engagement.

## Author contributions

S.N: contributed to conception and design, contributed to funding acquisition, analysis, and interpretation, drafted manuscript, and critically revised manuscript. J.G: contributed to conception and design, analysis, contributed to interpretation, drafted manuscript, and critically revised the manuscript. R.A: contributed to design, analysis, contributed to interpretation, drafted manuscript and critically revised manuscript and gave final approval. J.H: contributed to design, contributed to analysis and interpretation, drafted manuscript, and critically revised the manuscript and gave final approval. G.G: contributed to conception and design, contributed to interpretation, drafted manuscript, and critically revised the manuscript and gave final approval. K.K: contributed to conception and design, contributed to interpretation, drafted manuscript, and critically revised the manuscript and gave final approval. A.P: contributed to interpretation, drafted manuscript, and critically revised the manuscript and gave final approval L.J: contributed to conception and design, contributed to interpretation, drafted manuscript, and critically revised the manuscript and gave final approval.

## Ethics statement

Ethics approval for this study was granted by the Aboriginal Health Council of South Australia Human Research Ethics Committee (04-22-990) and the University of Adelaide Human Research Ethics Committee. Written informed consent was obtained from all participants prior to the interviews. Participants were informed about the study procedures, their voluntary participation and their right to withdraw at any time without consequences.

## Conflict of interest

The authors declare that they have no known competing financial interests or personal relationships that could have appeared to influence the work reported in this paper.

## Appendix Supplementary materials

Image, application 1
