# Corrigendum to ‘Psychosocial Impact of Dental Aesthetics Among Aboriginal and Torres Strait Islander peoples: Mixed-Methods Study Using Psychosocial Impact of Dental Aesthetics Questionnaire’ [International Dental Journal Volume 76, Issue 1, February 2026, 109339]

**DOI:** 10.1016/j.identj.2025.109384

**Published:** 2026-01-17

**Authors:** Sonia Nath, Johanna Groundwater, Ria Aiyar, Joanne Hedges, Gina Guzzo, Kostas Kapellas, Alexander Pham, Lisa Jamieson

**Affiliations:** Indigenous Oral Health Unit, Australian Research Centre for Population Oral Health, Adelaide Dental School, The University of Adelaide, South Australia, Australia

The authors regret that the current title is incorrect ‘Psychosocial Impact of Dental Aesthetics Among Aboriginal and Torres Strait Islanders: Mixed-Methods Study Using Psychosocial Impact of a Dental Aesthetics Questionnaire’. The correct title is ‘Psychosocial Impact of Dental Aesthetics Among Aboriginal and Torres Strait Islander peoples: Mixed-Methods Study Using Psychosocial Impact of Dental Aesthetics Questionnaire’

The authors would like to apologise for any inconvenience caused.

