# Supplementary material for: Psychosocial Impact of Dental Aesthetics Among Aboriginal and Torres Strait Islanders: Mixed‑Methods Study Using Psychosocial Impact of a Dental Aesthetics Questionnaire
Source: Int Dent J. 2025 Dec 15;76(1):109339. doi: 10.1016/j.identj.2025.109339 (PMC12767711; doi:10.1016/j.identj.2025.109339)
Supplement: Supplementary file 1 [file mmc1.docx]

Supplementary Files

Manuscript:

Psychosocial impact of dental aesthetics among Aboriginal and Torres Strait Islanders: mixed‑methods study using PIDAQ

Table of Contents

[Supplementary 1. CONSIDER STATEMENT 2](#_Toc213327901)

[Supplementary 2. Psychosocial Impact of Dental Aesthetics Questionnaire (PIDAQ) 7](#_Toc213327902)

[Scoring instructions: 8](#_Toc213327903)

[Brief validity assessment: 8](#_Toc213327904)

[Supplementary 3. The PIDAQ questionnaire 10](#_Toc213327905)

[Supplementary 4. Model-fit diagnostics for GLM PIDAQ outcomes 11](#_Toc213327906)

[Supplementary 5. Sensitivity analysis using OLS estimates for the total PIDAQ score with HC3 robust standard errors 12](#_Toc213327907)

[Reference: 13](#_Toc213327908)

## Supplementary 1. CONSIDER STATEMENT

| **Governance** | |
| --- | --- |
| 1. | *Describe partnership agreements between the research institution and Indigenous-governing organization for the research, (e.g., Informal agreements through to MOU (Memorandum of Understanding) or MOA (Memorandum of Agreement)).*   - Formal letters of support provided by Sovereign Aboriginal organisations that the chief investigator (LJ) and Senior Aboriginal Research Officer (JH) had connections with, including Chief Executive Officers and leaders within key South Australian Aboriginal Community Controlled Health Organisations (ACCHOs). |
| 2. | *Describe accountability and review mechanisms within the partnership agreement that addresses harm minimization.*   - Ethics approval granted by the Aboriginal Health Council of South Australia Human Research Ethics Committee and the University of Adelaide Human Research Ethics Committee. - Letters of support provided by CEOs of South Australian ACCHOs in relation to the research procedures, and protocols. - Study oversight was provided by an Indigenous Oral Health Unit Reference Group (IOHURG), which offered guidance across all project dimensions including staff recruitment, participant engagement, data collection protocols, analytical processes, and community feedback mechanisms. |
| 3. | *Specify how the research partnership agreement includes protection of Indigenous intellectual property and knowledge arising from the research, including financial and intellectual benefits generated (e.g., development of traditional medicines for commercial purposes or supporting the Indigenous community to develop commercialization proposals generated from the research).*   - Indigenous governance rights are protected by the partnership between the Indigenous Oral Health Unit and key ACCHOs. - Informed consent was obtained from all study participants prior to their involvement in the research. - The project received ongoing guidance from the IOHURG, the Senior Indigenous Research Officer, and ACCHO contacts. |
| **Prioritization** | |
| 4. | *Explain how the research aims emerged from priorities identified by either Indigenous stakeholders, governing bodies, funders, non-government organization(s), stakeholders, consumers, and empirical evidence.*   - An established body of evidence highlights poorer overall wellbeing and oral health the Indigenous Australian population compared to the non-Indigenous Australian population. - This includes a lack of culturally safe oral healthcare and several barriers to accessing dental services, as identified in the broader literature and by Indigenous leaders within South Australian ACCHOs. - The intervention aims included promoting: quality of life; self-confidence through supporting communities with dental aesthetics and function; productivity in school or workplaces; and better prognosis for chronic diseases linked with poor oral health. Qualitative understandings can shape health services planning and delivery of culturally safe dental care. |
|  | **Relationships** (Indigenous stakeholders/participants/research team) |
| 5. | *Specify measures that adhere and honor Indigenous ethical guidelines, processes, and approvals for all relevant Indigenous stakeholders, recognizing that multiple Indigenous partners may be involved, e.g., Indigenous ethics committee approval, regional/national ethics approval processes*.   - Ethics approval granted by the Aboriginal Health Council of South Australia Human Research Ethics Committee and the University of Adelaide Human Research Ethics Committee. - Research aims and protocols reviewed and endorsed by the ACCHOs and Aboriginal health services involved, and the IOHURG. |
| 6. | *Report how Indigenous stakeholders were involved in the research processes (i.e., research design, funding, implementation, analysis, dissemination/recruitment).*   - Research design and implementation informed by the team’s Senior Indigenous Research Officer as well as Indigenous academics and the IOHURG. - Ongoing and longstanding relationship between the Indigenous Oral Health Unit, ACCHOs and Aboriginal health services who supported with dissemination and recruitment. - Study oversight was provided by an Indigenous Oral Health Unit Reference Group (IOHURG), which offered guidance across all project dimensions including staff recruitment, participant engagement, data collection protocols, analytical processes, and community feedback mechanisms. |
| 7. | *Describe the expertise of the research team in Indigenous health and research*   - The Indigenous and non-Indigenous research team have over 20 years combined experience working with Indigenous communities in the oral health field. - The research team were guided by Senior Aboriginal Research Officer, Ms Joanne Hedges (Yamatji). Ms Hedges has extensive experience in the implementation, collection, and analysis of Indigenous health data, including leading a longitudinal study exploring oral HPV and oropharyngeal cancer prevalence in over 1000 Indigenous Australian adults. - Chief Investigator Prof Lisa Jamieson is a dentist with a strong focus on Indigenous oral health and Indigenous research engagement, embedded in a world-class research centre (Indigenous Oral Health Unit), with strong connections to Aboriginal health service providers and policy makers. - Along with oral health therapist, Dr Kostas Kapellas, Prof Jamieson and Dr Kapellas have led and conducted various interventions involving comprehensive dental care for Indigenous Australians, including collection of biodata to assess clinical biomarkers before and after dental intervention. - Dr Sonia Nath is a dentist with over 5 years of experience engaging with Indigenous communities through culturally safe oral health research practices. This includes leading the current project providing culturally safe dental care for over 400 Indigenous Australian participants, overseeing and conducting clinical examinations for Indigenous communities in metropolitan and regional South Australia. - Dr Ria Aiyar has sound experience in conducting qualitative research with diverse groups, including Indigenous communities in relation to health systems and wellbeing. Dr Aiyar has been involved in leading the 60-month follow-up phase of the longitudinal study exploring oral HPV and oropharyngeal cancer in Indigenous Australian adults residing in Metropolitan Adelaide. - Dr Gina Guzzo is a microbiome scientist with extensive experience in the coordination of fieldwork, dental charting, microbial sample collection, point-of-care testing, and oral health communication with Indigenous study participants and community members in metropolitan and regional communities. - Dr Alexander Pham is a dentist experienced in Indigenous oral health research. As part of various projects conducted at the Indigenous Oral Health Unit, Dr Pham has travelled to regional and remote Indigenous communities to provide dental screening for children, and as part of the current study, for children and adults. - Ms Johanna Groundwater is an Oral Health Therapist with experience in Indigenous research and treating Indigenous community regionally and remotely. |
|  | **Methodology** |
| 8. | *Describe the methodological approach of the research including a rationale of methods used and implication for Indigenous stakeholders, e.g., privacy and confidentiality (individual and collective)*   - Mixed method approach:   (1) Culturally adapted Psychosocial Impacted of Dental Aesthetics Questionnaire (PIDAQ) used to quantify the impacts of dental self-confidence, social impact, psychological impact and aesthetic concern on individual’s social and emotional wellbeing.  (2) Reflexive thematic analysis was used to analyse the qualitative data, as this approach allowed us to centre the lived experiences of participants in terms of their wellbeing and oral health experiences.   - All qualitative and quantitative was de-identified to ensure privacy and confidentiality. - The Senior Aboriginal Research Officer provided ongoing guidance and input throughout data generation and analysis. |
| 9. | *Describe how the research methodology incorporated consideration of the physical, social, economic and cultural environment of the participants and prospective participants. (e.g., impacts of colonization, racism, and social justice). As well as Indigenous worldviews.*   - The research centred health equity in considering the impacts of colonisation, racism and social justice on participants’ oral health and healthcare experiences. - The social and emotional wellbeing model and its seven domains were considered within the current research praxis, stemming from Indigenous understandings and approaches to wellbeing. In this way, health is viewed as holistic, the importance of kinship is recognised, and Aboriginal and Torres Strait Islander strengths are centred. |
|  | **Participation** |
| 10. | *Specify how individual and collective consent was sought to conduct future analysis on collected samples and data (e.g., additional secondary analyses; third-parties accessing samples (genetic, tissue, blood) for further analyses).*   - Data that is published is summary data and is de-identified. - Data is securely stored and kept according to ethics protocols, with all access logged requiring multi-factor authentication and limited to research staff. - Participants involved in the research own the data. |
| 11. | *Described how the resource demands (current and future) placed on Indigenous participants and communities involved in the research were identified and agreed upon including any resourcing for participation, knowledge, and expertise.*   - In limiting the limited resource demands placed on participants, participants could partake in oral epidemiological examinations at a date, time, and location of their choosing (e.g., their home, a community centre, a University research facility). - Participation in qualitative interviews was voluntary and participants could withdraw at any time during the follow-up. The option to complete the interview over the phone at a later time was also offered. |
| 12 | *Specify how biological tissue and other samples including data were stored, explaining the processes of removal from traditional lands, if done, and of disposal.*   - Point-of-care testing in collecting biodata from Indigenous participants provided participants with immediate results, was less invasive requiring small volumes of blood and urine and did not require storage or involvement of third-party laboratory services. - Qualitative and quantitative data is securely stored and kept according to ethics protocols with all access logged requiring multi-factor authentication and limited to research staff. |
|  | **Capacity** |
| 13. | *Explain how the research supported the development and maintenance of Indigenous research capacity (e.g., specific funding of Indigenous researchers).*   - The research was guided and led by project investigator and Senior Aboriginal Research Officer (JH). Research Officers were trained by the Senior Aboriginal Research Officer and Chief Investigator in oral health promotion, and by the microbiome scientist (GLG) in the use of point-of-care testing. - Senior Aboriginal Research Officer provided ongoing guidance to non-Indigenous members of the research team regarding culturally safe practices. |
| 14. | *Discuss how the research team undertook professional development opportunities to develop the capacity to partner with Indigenous stakeholders?*   - All researchers engaged in education regarding culturally safe research practices, as well as ongoing discussions around talking about oral health and wellbeing with Indigenous communities. These development opportunities were led by the Senior Aboriginal Research Officer. - The Senior Aboriginal Research Officer has extensive experience providing education and guidance to both Indigenous and non-Indigenous researchers regarding safe research practices, as well as information around Indigenous oral health and wellbeing. |
|  | **Analysis** **and** **interpretation** |
| 15. | *Specify how the research analysis and reporting supported critical inquiry and a strength-based approach that was inclusive of Indigenous values.*   - Analysis reflected the positive outcomes the intervention had in terms of addressing aims around promoting functional aspects of oral health, supporting chronic disease management, and addressing inequities in health and healthcare. - Additionally, the social and emotional wellbeing model and its seven domains were considered within the current research praxis, whereby Aboriginal and Torres Strait Islander strengths were centred, and health viewed holistically. |
|  | **Dissemination** |
| 16. | *Describe the dissemination of the research findings to relevant Indigenous governing bodies and peoples.*   - The study team disseminated research update newsletters to all participants, and Indigenous stakeholder groups including the involved ACCHOs. Results will also be published in academic journals. - Findings will also be shared with key stakeholders at Indigenous health and oral health conferences held nationally and internationally. |
| 17. | *Discuss the process for knowledge translation and implementation to support Indigenous advancement (e.g., research capacity, policy, investment).*   - The project has various important outcomes, including empirical evidence of how culturally safe dental care can promote understandings regarding prognosis for chronic diseases linked with poor oral health, as well as how this can influence social and emotional wellbeing for Indigenous Australians. This will be important for health services planning, especially in the ACCHO sector, where the management of dental diseases in a culturally safe manner for better chronic disease outcomes is currently insufficiently understood, planned and budgeted for. |

## Supplementary 2. Psychosocial Impact of Dental Aesthetics Questionnaire (PIDAQ)

The short version of PIDAQ was used to assess the psychosocial effects of dental appearance among Indigenous Australian adults. The short version of PIDAQ included eight items, each mapped to four domains: Dental Self-Confidence (2 items), Social Impact (2 items), Psychological Impact (2 items), and Aesthetic Concern (2 items).^1, 2^ This tool was culturally adapted, and all the questions were worded in a positive frame. All items were reworded in collaboration with Indigenous advisors to ensure cultural relevance and a strengths-based focus, while retaining the original conceptual structure. In the Dental Self‑Confidence domain, items were retained unchanged. In the Psychosocial Impact domain, negative-based items (e.g., “I wish my teeth looked better”) were reworded into strengths-based statements (e.g., “I like the way my teeth look”), and “Most people have nicer teeth than I do” became “I feel like I have nice teeth compared to others”. Social Impact items were reframed to emphasise autonomy and comfort with visibility of teeth with “When I talk, smile or laugh I like to show my teeth” and to reduce anxiety about external judgments by stating “I am not concerned about what others think about my teeth.” In the Aesthetic Impact domain, aesthetic items were also adjusted to emphasise autonomy and cultural traditions, including new culturally specific options such as choosing not to be photographed or recognition of community lore around teeth.

The full adapted item set is described below, including both the original and modified item wording:

| **Domain** | **Original item** | **Modifications/Adapted item** |
| --- | --- | --- |
| **Dental self confidence** | I am proud of my teeth | I am proud of my teeth |
|  | I like to show my teeth when I smile. | I like to show my teeth when I smile. |
| **Psychosocial Impact** | I wish my teeth looked better | I like the way my teeth look |
|  | Most people have nicer teeth than I do | I feel like I have nice teeth compared to others |
| **Social Impact** | I hold myself back when I smile so my teeth don’t show so much | When I talk, smile or laugh I like to show my teeth. |
|  | I sometimes worry about what members of the opposite sex think about my teeth. | I am not concerned about what others think about my teeth |
| **Aesthetic Concern** | I don’t like to see my teeth in the mirror | I like to see my teeth in the **mirror**. |
|  | I don’t like my own teeth in photos | For cultural reasons I choose not to have my photo taken.  Yes/No  I don’t mind showing my teeth in **photos**. |

### Scoring instructions:

All items were scored on a five-point Likert scale (1 = “Strongly disagree”, 5 = “Strongly agree”), with higher scores consistently reflecting a negative psychosocial impact of dental aesthetics. No reverse-coding was required as all items were phrased positively. The possible total score ranged from 8 to 40, with each subscale yielding a range from 2 (lowest) to 10 (highest).

### Brief validity assessment:

Face and content validation were done. To check for the reliability, the intraclass correlation coefficient (ICC) was calculated among 20 sample populations that were not part of the main study. The overall Cronbach α was 0.91.

**Reliability of the score of the culturally adapted PIDAQ**

| **Items** | **Cronbach’s α** (overall:0.92) |
| --- | --- |
| Look | 0.90 |
| Smile | 0.90 |
| Show | 0.91 |
| Proud | 0.91 |
| Mirror | 0.91 |
| Nice | 0.91 |
| Photo | 0.92 |
| Worry | 0.93 |

## Supplementary 3. The PIDAQ questionnaire

| **THESE QUESTIONS are about the appearance of your teeth as they are now, and how they affect you overall.** | | | | | | |
| --- | --- | --- | --- | --- | --- | --- |
| 1. In my Community/mob, we respect traditions and customs related to cultural lore with teeth. | Yes  □1 | | No  □2 | | Not applicable (NA)  □3 | |
|  | **Strongly Agree** | **Agree** | **Neither agree nor disagree** | **Disagree** | | **Strongly disagree** |
| 2. I am **proud** of my teeth. | □_1_ | □_2_ | □_3_ | □_4_ | | □_5_ |
| 3. I like to show my teeth when I smile. | □_1_ | □_2_ | □_3_ | □_4_ | | □_5_ |
| 4. I like the way my teeth **look**. | □_1_ | □_2_ | □_3_ | □_4_ | | □_5_ |
| 5. I feel like I have **nice** teeth compared to others. | □_1_ | □_2_ | □_3_ | □_4_ | | □_5_ |
| 6. When I talk, smile, or laugh, I like to **show** my teeth. | □_1_ | □_2_ | □_3_ | □_4_ | | □_5_ |
| 7. I am not worried about what **others** think about my teeth. | □_1_ | □_2_ | □_3_ | □_4_ | | □_5_ |
| 8. I like to see my teeth in the **mirror**. | □_1_ | □_2_ | □_3_ | □_4_ | | □_5_ |
| 9A. For cultural reasons, I choose not to have **my photo taken.** | Yes  □1 | | No  □2 | | | Not applicable (NA)  □3 |
| 9B. don’t mind showing my teeth in **photos**. | □_1_ | □_2_ | □_3_ | □_4_ | | □_5_ |

## Supplementary 4. Model-fit diagnostics for GLM PIDAQ outcomes

| Outcome | Metric | Value |
| --- | --- | --- |
| PIDAQ total | Pearson dispersion | 0.09 |
| PIDAQ total | Deviance over df | 0.10 |
| Dental self confidence | Pearson dispersion | 0.14 |
| Dental self confidence | Deviance over df | 0.16 |
| Social impact | Pearson dispersion | 0.13 |
| Social impact | Deviance over df | 0.15 |
| Psychological impact | Pearson dispersion | 0.12 |
| Psychological impact | Deviance over df | 0.14 |
| Aesthetic concern | Pearson dispersion | 0.09 |
| Aesthetic concern | Deviance over df | 0.11 |

Pearson dispersion is the Pearson chi‑square divided by residual degrees of freedom. Deviance over df is the residual deviance divided by residual degrees of freedom.

Diagnostics were calculated for each generalised linear model (Gamma, log link) fitted to the total and subscale PIDAQ outcomes; complete‑case analyses were used.

## Supplementary 5. Sensitivity analysis using OLS estimates for the total PIDAQ score with HC3 robust standard errors

| term | Effect | LCI | UCI | *p value* | P adj BH |
| --- | --- | --- | --- | --- | --- |
| Age category: >55 years | -1.27 | -4.32 | 1.79 | 0.42 | 0.50 |
| Age category: 35-54 years | -3.32 | -6.18 | -0.46 | 0.02 | 0.09 |
| Sex: Female | -0.14 | -2.57 | 2.29 | 0.91 | 0.91 |
| Location: Regional/Remote | 1.92 | -1.25 | 5.10 | 0.24 | 0.35 |
| Education: High school or less | -0.70 | -3.06 | 1.66 | 0.56 | 0.61 |
| **Household size: >4 people** | -2.26 | -5.06 | 0.53 | 0.11 | 0.26 |
| Healthcare card: Yes | 1.34 | -0.95 | 3.63 | 0.25 | 0.35 |
| Last dental visit: More than one year ago | 1.40 | -0.93 | 3.72 | 0.24 | 0.35 |
| **Reason for last dental visit: Problem** | 4.30 | 1.58 | 7.02 | 0.00 | 0.02 |
| **Avoided dental visit due to cost: Yes** | 1.92 | -0.56 | 4.40 | 0.13 | 0.26 |
| **Difficulty in paying $100 for dental treatment: Hard** | 1.02 | -1.50 | 3.53 | 0.43 | 0.50 |
| **Self-rated general health: Poor** | 3.18 | 0.57 | 5.78 | 0.02 | 0.08 |
| **Self-rated oral health: Poor** | 2.54 | -0.27 | 5.36 | 0.08 | 0.22 |

LCI: Lower confidence interval; UCI: Upper confidence interval; P adj BH: Benjamini–Hochberg adjusted p-value. A p-value of less than 0.05 was considered significant.

Ordinary Least Squares (OLS) model included all listed covariates; missing observations were excluded via complete‑case analysis.

Reference categories: 18–34 years (age), Male (sex), Major cities (location), Tertiary education (education), household size 0–4, No healthcare card, last dental visit <1 year, Check‑up (reason), No avoidance due to cost, Not hard to pay AUD$100, Good self‑rated general health, and Good self‑rated oral health.

## Reference:

1. Wan Hassan WN, Makhbul MZM, Othman SA, Yusof ZYM. Validation of the Simplified Malaysian Psychosocial Impact of Dental Aesthetics Questionnaire for the Sociodental Approach to Estimate the Orthodontic Treatment Need. Int J Environ Res Public Health 2022;19.

2. Alsanabani AAM, Yusof ZYM, Wan Hassan WN, Aldhorae K, Alyamani HA. Short Versions of the Arabic Psychosocial Impact of Dental Aesthetics Questionnaire for Yemeni Adolescents: Cross-Sectional Derivation and Validation. Children (Basel) 2022;9.
